# Successful closed manipulation of a pure lateral traumatic dislocation of the elbow joint using a modified Stimson's technique: a case report

**DOI:** 10.1186/1752-1947-2-170

**Published:** 2008-05-22

**Authors:** Sameer K Khan, Rajat Chopra, Debasis Chakravarty

**Affiliations:** 1 Department of Trauma and Orthopaedics, Peterborough District Hospital, Peterborough and Stamford Hospitals NHS Foundation Trust, Peterborough, PE3 6DA, Cambridgeshire, UK

## Abstract

**Introduction:**

Pure lateral elbow dislocation is rare, and a successful closed reduction is even rarer. Reduction can be hindered by swelling, soft tissue interposition or associated fractures.

**Case presentation:**

We present a pure lateral traumatic dislocation of the elbow joint in a 40-year-old man. This was successfully manipulated and reduced in casualty using a modification of the gravity-aided 'hanging arm' technique originally described for shoulder dislocations by Stimson.

**Conclusion:**

We strongly recommend the use of this simple technique in these rare yet difficult injuries, in order to avoid potential complications with general anaesthesia and surgery.

## Introduction

Pure lateral elbow dislocation is rare, and a successful closed reduction is even rarer. Reduction can be hindered by swelling, soft tissue interposition or associated fractures. The elbow dislocation of the case we present here was irreducible by conventional methods, so we adapted a modification of a historical method to successfully reduce it. A historical review is discussed subsequently. To the best of our knowledge, this is the first reported application of this particular technique for this rare injury.

## Case presentation

**Figure 1:**
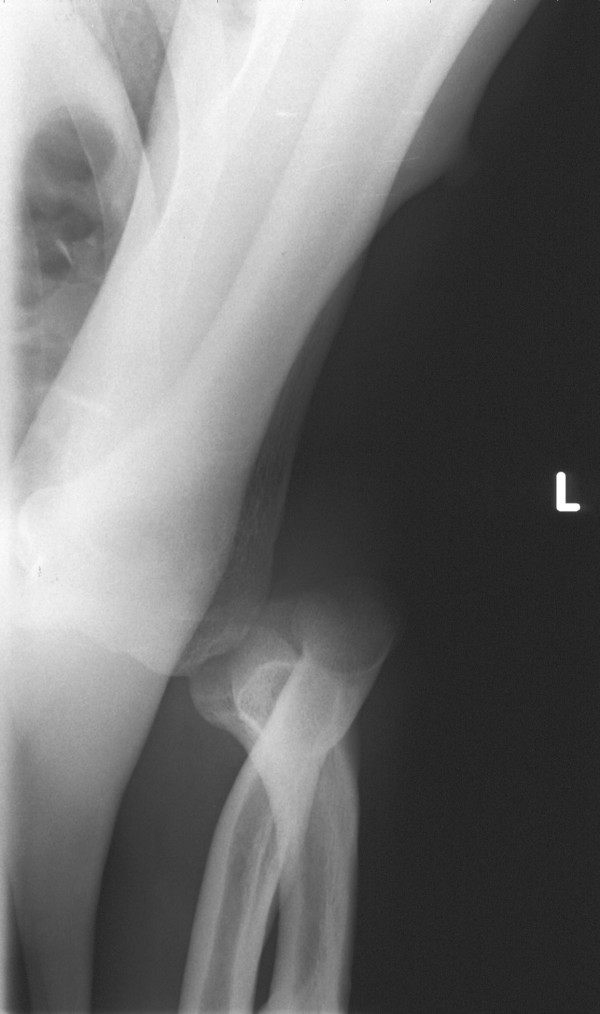
Anteroposterior view showing pure lateral elbow dislocation.

A 40-year-old right-hand-dominant man was offloading beer crates while perched on a box. As he turned round, he lost his balance and fell with his left hand outstretched and elbow extended. He presented with a swollen and deformed elbow joint. It was held in 60° of flexion, and with the forearm in pronation. Distal circulation and motor function were intact, but he complained of pins and needles in the ulnar nerve distribution. His radiographs showed a true lateral displacement of the left proximal radius and ulna in relation to the humerus (Figure 1). The olecranon was in contact with the lateral condyle, and in line with the transverse axis of the distal end of the humerus. However, the anatomical relationship of the radius and ulna was maintained and no fractures could be visualised.

A closed reduction was attempted under light sedation by the casualty registrar using conventional methods, but proved unsuccessful. The patient was re-examined by the orthopaedic team and was placed prone with the affected left arm hanging by the edge of the bed. Ten minutes later, with the patient still sedated, the elbow was first disimpacted by applying longitudinal traction on the forearm with counter-traction on the arm. It was then enlocated applying gentle medial pressure on the olecranon. Valgus and varus instability of the elbow was checked in full extension and 30° of flexion. Normal sensation returned spontaneously in the ulnar nerve distribution.

**Figure 2:**
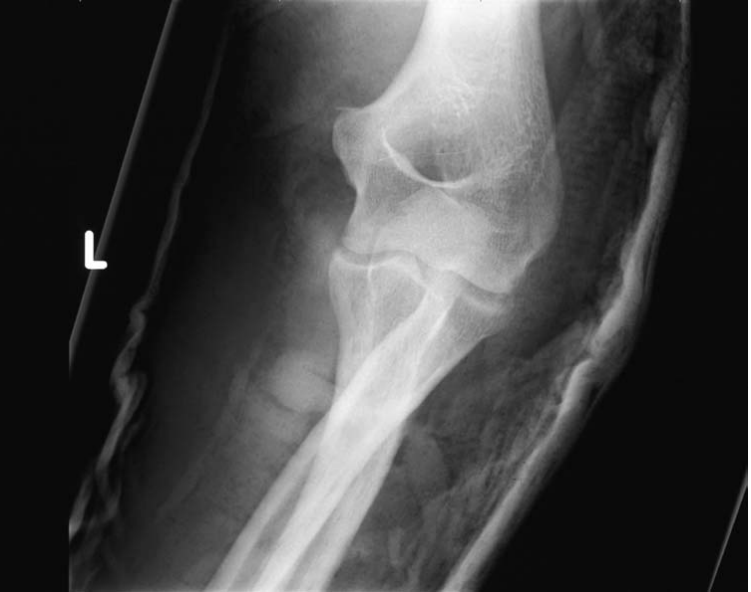
Anteroposterior film confirming that the elbow is reduced.

**Figure 3:**
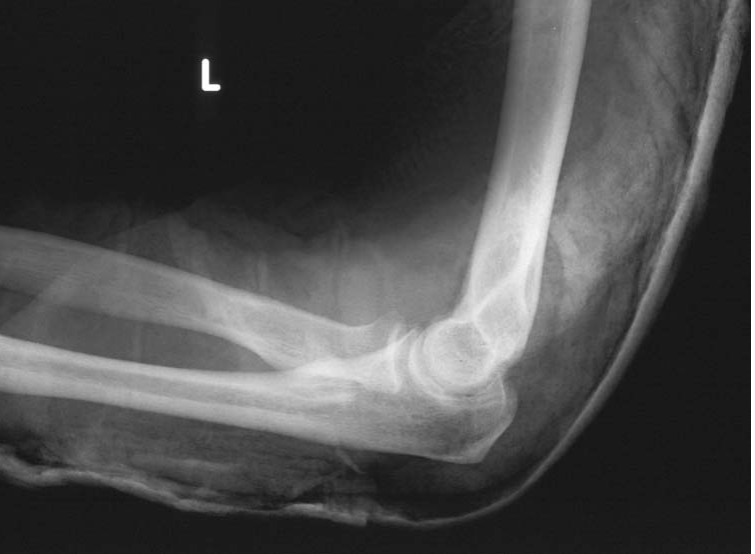
Lateral film showing the reduced elbow joint in profile.

The elbow was then immobilised in plaster in 90° of flexion. Radiographs confirmed a satisfactory reduction with normal joint congruity (Figures 2 and 3). The patient was offered a follow-up appointment a week later, but could attend only 3 weeks later on account of personal commitments. His plaster was removed and his elbow stressed to check for medial or lateral ligamentous instability. Nothing untoward was found, but he was started on an intensive physiotherapy regime. He subsequently regained the normal range of elbow movements and was discharged from care after his second follow-up visit at 2 months.

## Discussion

Stimson [1] originally described his 'hanging arm' technique for reducing shoulder dislocations. This method consisted of the patient lying prone in a canvas cot, with the affected arm hanging through a hole in the canvas. Weights were then placed in the dependent hand, and reduction occurred due to the synergistic effect of muscle relaxation and gravity. Rollinson [2] modified this technique by hanging the affected arm over the side of the bed. Also, instead of applying weights he used a supraclavicular brachial nerve block to relieve pain and evoke muscle relaxation.

Levine [3] described reduction of an elbow dislocation by seating the patient in a chair, with the arm dangling over the back of the chair. This however required co-operation from the patient, which can be difficult in certain circumstances. Parwin [4] has employed a technique similar to Stimson's, consisting of prone positioning, traction at the wrist, and elevation of the humerus to produce reduction. Meyn and Quigley [5] improved on this by grasping the olecranon with the operator's other hand, and guiding it into place. Prone positioning and gravity-aided traction has also been used successfully by Minford and Beattie [6]. All of these techniques have been employed to reduce posterior dislocations, contrary to the pure lateral in this case. Our technique also differs in requiring two persons to manipulate, as we feel that guiding the olecranon in a purely medial direction requires both hands from the main operator, with the assistant constantly applying counter-traction. The ease of reduction justifies the use of an additional pair of hands.

Simple lateral dislocations should theoretically be amenable to closed manipulation, as documented by Vijaya [7] in his case report. However, reduction can be impeded by fractured articular fragments or interposed muscle. Exarchou [8] found the anconeus muscle interposed between the articular surfaces and preventing reduction. Vaidya [9] has documented the brachialis muscle and a coronoid chip fracture to be the causes of the irreducibility in their patient. Both these cases required open reduction and stabilisation.

## Conclusion

Pure lateral elbow dislocation is rare, and a successful closed reduction is even rarer. The reduction can prove difficult, even under general anaesthesia, due to swelling, soft tissue interposition or associated fractures. We have adapted a modification of Stimson's original method to reduce a purely lateral elbow dislocation. Such an atraumatic and mechanically simple technique can prove very useful in a busy casualty department. It also avoids potential complications with general anaesthesia and surgery, while giving an anatomically congruent reduction.

## Competing interests

The authors declare that they have no competing interests.

## Authors' contributions

SK and RC applied this method to reduce this patient's elbow joint. DC followed him up in clinic with check radiographs. All authors undertook the literature search and preparation of the manuscript. All authors read and approved the final manuscript.

## Consent

Written informed consent was obtained from the patient for publication of this case report and accompanying images. A copy of the written consent is available for review by the Editor-in-Chief of this journal.
